# Editorial: Adaptation to Psychological Stress in Sport

**DOI:** 10.3389/fpsyg.2020.02199

**Published:** 2020-09-08

**Authors:** Martin J. Turner, Marc V. Jones, Anna C. Whittaker, Sylvain Laborde, Sarah Williams, Carla Meijen, Katherine A. Tamminen

**Affiliations:** ^1^Department of Psychology, Manchester Metropolitan University, Manchester, United Kingdom; ^2^Faculty of Health Sciences, Sport University of Stirling, Stirling, United Kingdom; ^3^School of Sport, Exercise & Rehabilitation Sciences, University of Birmingham, Birmingham, United Kingdom; ^4^Department of Performance Psychology, Institute of Psychology, German Sport University Cologne, Cologne, Germany; ^5^Faculty of Sport, Health and Applied Science, St Mary's University, Twickenham, United Kingdom; ^6^Faculty of Kinesiology and Physical Education, University of Toronto, Toronto, ON, Canada

**Keywords:** stress, psychophysiology, pressure, emotion, sport

Psychological stress is ubiquitous in sport. Unsurprisingly then, research that examines the antecedents, correlates, consequences, and interventions pertaining to psychological stress in sport is sizable and broad. With this Research Topic we aimed to capture the breadth and depth of work taking place around the theme of adaptation to psychological stress in sport. Pleasingly, 111 authors responded to our call for papers, contributing 25 papers between them. In this Editorial we undertake the difficult task of synthesizing these contributions, and highlight important implications that could influence future research and practice.

One thing that is clear from this Research Topic, is that adaptation to psychological stress is truly a biopsychosocial phenomenon. Whether papers explore biological (bio), psychological (psycho), or social constructs, or a combination of the three, any collective conclusions drawn from the contributions here must involve an appreciation of all three facets. The multiple ways in which these facets interact to predict and impact upon affective and behavioral outcomes within a sport setting is complex, which is one of the reasons we do not have a single unified way of understanding adaptation to psychological stress. In this Research Topic we have some useful theories that capture this biopsychosocial perspective, including two revised theories of challenge and threat states in sport. Indeed, numerous papers within this Research Topic align with and draw upon challenge and threat theory.

Britton et al. provide an important piece of work that speaks to the complex interaction of constructs that are implicated within challenge and threat theory. They recruited adolescent athletes who completed self-reported stress reactivity and cognitive appraisals on approach to competition and a retrospective assessment of emotions, coping strategies, and subjective performance. The path analysis revealed that perceived stress reactivity had direct and indirect effects on the appraisal of higher stressor intensity, lower perceived control, higher perceived threat, negative emotions, and maladaptive coping. Increased threat, positive and negative emotions, and maladaptive coping were associated with performance satisfaction. The complex interaction of cognitive appraisal constructs and affect is also captured by Harwood et al. in their study of stress among parents of competitive British tennis players, in which they consider the primary appraisals, emotions, and coping strategies associated with self-disclosed stressors. The mixed methods analyses showed that a range of organizational, competitive, and developmental stressors were predominantly appraised as harm or challenge, and that anxiety and anger were the most prominent emotions experienced by parents. In particular, parents experienced greater anger in relation to competition (compared to organizational and developmental) stressors, harm appraisal increased negative emotions, and challenge appraisal increased positive emotions.

Where most challenge and threat research has focused on singular events, Moore et al. focused on examining the generalizability of challenge (adaptive) and threat (maladaptive) by examining the consistency of challenge and threat evaluations across potentially stressful situations. In their sample of roller derby players, they found some idiosyncrasies in the athletes' tendency to view particular stressors as more of a challenge or threat. A key take away message from this paper is that there is an interaction between the person and the situation in determining challenge and threat, a notion at the heart of transactional stress theories such as cognitive appraisal theory (Lazarus and Folkman, 1984), and rational emotive behavior therapy (REBT)—also featured in this Research Topic.

The interacting constructs and the personal-situational variability of cognitive appraisals, and by extension challenge and threat, provides an exciting task for researchers to conceptualize challenge and threat in testable theories. This Research Topic contains two pieces of work that seek to adjust and extend theory, that of Uphill et al. and Meijen et al.. Both reflect a re-conceptualization of challenge and threat theory applied to sport. Uphill et al. provide a critical review of challenge and threat literature, and propose a new theory, Evaluative Space Approach to Challenge and Threat (ESACT). The ESACT reconciles some of the ambiguities found in the extant research and draws upon the Evaluative Space Model (ESM). One of Uphill's suggestions is that rather than seeing challenge and threat as opposite ends of a single bipolar continuum, it might be better to consider that individuals could be (1) challenged, (2) threatened, (3) challenged and threatened, or (4) neither challenged or threatened by a particular stimulus. The article by Meijen et al. also offers a rethink of the dichotomous nature of challenge and threat but is more conservative than the suggestions of Uphill et al. Meijen et al. provide a review and revision of the Theory of Challenge and Threat States in Athletes (TCTSA), with a specific focus on the predictions made in the TCTSA and inclusion of Lazarusian cognitive appraisal constructs. The revised TCTSA (TCTSA-R) considers additional biomarkers of challenge and threat, includes more specific predispositional factors that influence challenge and threat, and offers a more parsimonious integration of Lazarusian ideas of cognitive appraisal and challenge and threat. Most notably, Meijen et al. propose a 2 × 2 bifurcation theory of challenge and threat, which reflects a polychotomy of four states: high challenge, low challenge, low threat, and high threat. For example, in low threat, an athlete can evince a threat state but still perform well so long as they perceive high resources. We urge the research community to test the hypotheses posited by Uphill et al. and Meijen et al. to progress this area.

The TCTSA-R places a greater emphasis on dispositional factors compared to the original TCTSA, but this aspect of challenge and threat theory is somewhat underdeveloped. One factor that may predispose athletes to threat is the extent to which they hold irrational beliefs, a notion examined in Chadha et al., in which path analyses across two study phases revealed how cognitive appraisals, irrational beliefs, and challenge and threat co-occur to predict affective states among golfers, such that golfers who reported more negative cognitive appraisals and higher irrational beliefs, were more likely to report greater threat, and subsequent higher anxiety and negative affect, and a less facilitative interpretation of their anxiety symptoms for performance. This offered some theoretical advancement to both theories of challenge and threat, and Rational Emotive Behavior Therapy. Similarly, exploring dispositional traits that could affect performance under pressure, Clarke et al. examined personality traits in predicting yips and choking susceptibility in a group of golfers and archers. They found that 11 variables correctly classified 71% of choking and non-choking participants and that a combination of four variables correctly classified 69% of the yips and non-yips affected participants. Notably, conscientiousness and private self-consciousness were the largest contributors to the choking model, whilst conscientiousness and perfectionistic self-promotion were the largest contributors to the yips model. Another dispositional trait relevant to challenge and threat is rumination which is addressed by Kröhler and Berti who used data from 157 competitive athletes from different sports to demonstrate that sports and competition-related ruminative mechanism exists and further that ruminative cognitions are related to the cognitive basis of state orientation. In another study of personality traits, Frenkel, Brokelmann et al. set out to identify protective factors in stressful situations in risk sports. Specifically, the authors experimentally examined the role of sensation seeking and dispositional mindfulness on the stress response to a risk sport-specific stressor; the Heidelberg Risk Sport-Specific Stress Test (HRSST–evaluated in the Research Topic in an additional paper by Frenkel, Laborde et al.). Their results indicate that high sensation seekers perceived the stressor as less stressful, but dispositional mindfulness did not predict anxiety.

Where irrational beliefs and rumination can predispose one to threat, one construct that could be an important protective factor from the negative impact of psychological stress is resilience. Hrozanova et al. reason that stress can deleteriously affect sleep, and that potentially mental resilience may protect individuals against the detrimental effects of stress on sleep. In their study, the authors investigated the effects of mental resilience, emotional (negative affect) and cognitive (worry) reactions to stress, and perceived stress, on the sleep quality of junior athletes. Results revealed that sleep quality was predicted by greater mental resilience sub-components Social Resources and Structured Style, and lower worry and perceived stress. Hrozanova et al. suggest that close attention should be paid to athletes' abilities to manage worry and perceived stress, and that mental resilience could act as a protective factor preventing sleep deterioration. Relevant to the notion of protective factors, some researchers have suggested that individual's histories of adversity may influence stress reactivity, an idea examined by Wadey et al. in their multi-study paper. The authors draw upon prominent sport injury, and challenge and threat theory to examine whether preinjury adversity affects postinjury responses over a 5-year period. They found that injured athletes with moderate preinjury adversity experienced less negative psychological responses and used more problem- and emotion-focused coping strategies compared to low or high preinjury adversity groups. In a follow-up study, Wadey et al. found that athletes with high preinjury adversities were excessively overwhelmed to the point that they were unable to cope with injury, while those with low preinjury adversities had not developed the coping abilities and resources needed to cope postinjury.

As previously stated in this editorial, adaptation to psychological stress is a biopsychosocial phenomenon, and thus, it is pleasing to see works included in the Research Topic that take a psychophysiological perspective on psychological stress. MacDonald and Wetherell assessed competitive anxiety and salivary diurnal cortisol in elite rowers during two training and two competition weekends. They found that anxiety levels were significantly greater during the competition phase compared with training, and specifically that cognitive anxiety was greater on the day of competition compared with the preparation day. They also found that the cortisol awakening response (CAR) magnitude was significantly reduced during the competition phase compared with training, with no differences between preparation and event days. Importantly, the findings indicate maladaptive responding during a period where maximized functioning is critical, whereby reduced or blunted CARs are typical in chronically stressed populations. Similarly examining acute psychophysiological responses, Guo et al. examined the impact of high and low coping self-efficacy (CSE) on the neural activity of athletes' cerebral cortex under acute psychological stress. Results indicate that high CSE athletes were better able to cope with the acute stressor, adjust their behaviors in a timely manner according to the results of their coping, and focus more on processing positive information, demonstrating significantly lower N1 amplitude and significantly shorter N1 latency, compared to low CSE athletes. In contrast to MacDonald and Wetherell, and Guo et al., Roberts et al. studied the longitudinal patterns of change in stress variables in the lead up to, during, and following the Invictus Games, in a cohort of wounded, injured, and sick military veterans. In addition, the interactions between psychosocial variables and salivary biomarkers of stress, and how these relate to veterans' health, well-being, illness, and performance, was investigated. Multilevel growth curve analyses revealed significant changes in growth trajectories of stress-related variables, with for example, anger and dejection emotions increasing, whilst challenge appraisals and excitement and happiness emotions decreased over the same timeframe. Alongside additional self-report effects (e.g., threat appraisals were found to negatively relate to performance, well-being, and mental health), the authors also found that organizational stressor intensity was positively related to cortisol exposure at competition. Collectively, the papers by MacDonald and Wetherell, Guo et al., and Roberts et al., lend additional support for the transactional nature of psychological stress.

There are a number of papers in the Research Topic that have significant theoretical and practical implications for adaptation to psychological stress in sport. In addition, there are number of papers included in the Research Topic that expressly posit potential interventions for successful adaptation. In one study, Quinton et al. examined whether mastery imagery ability was associated with stress response changes to a competitive car racing stress task following an imagery intervention. They also assessed the effects of different guided imagery content on pre-task cognitive and emotional responses. Based on the study results, the authors suggest that positive mastery imagery ability may act as a buffer against the stress effects of negative images. Imagery featured as part of the intervention tested in the Olmedilla et al. paper, whereby a program based on cognitive-behavioral therapy was applied with youth soccer players. Pre to post-test data demonstrated that athletes improved their stress management, and enhanced the use of psychological resources and techniques. One psychological intervention that has particular efficacy in endurance sports is action monitoring and this was explored by Vitali et al.. That is, to deal with discomfort, fatigue, and pain associated with endurance performance under pressure, athletes tend to direct attention to both internal (e.g., bodily) sensations and external (e.g., environmental) stimuli. Thirty-two male participants completed a time-to-exhaustion running task on a treadmill. There was no difference in performance regardless of the type or level of action monitoring employed.

One technique for which research evidence has been growing is mindfulness, which is at the center of the study by Shannon et al.. The authors posit that mindfulness training could be beneficial for athlete well-being, reducing stress, and increasing competence in mental health self-management. Indeed, their findings demonstrate that mindfulness training was directly related to positive changes in competence, resulting in indirect effects on mindfulness awareness, stress, and well-being, bringing into focus self-determination theory in athlete adaptation to psychological stress. Controlled breathing is often an important part of mindfulness and Laborde et al. explored slow-paced breathing (SPB) in two experiments. Both experiments involved SPB done either before (experiment 1) or after (experiment 2) 5 min of physical exercise (burpees). In both experiments, adaptation to psychological stress was investigated with a Stroop task, a measure of inhibition, which followed physical exercise. The results suggest that SPB realized before or after physical exercise has a positive effect regarding adaptation to psychological stress and specifically inhibition, however, the underlying mechanisms require further investigation. Another burgeoning literature within sport is the research concerning self-compassion. Ceccarelli et al. investigated the influence of self-compassion on athletes' psychological and physiological responses when recalling a sport failure. Athletes imagined past performance failure whilst a range of psychophysiological data were collected. Self-compassion positively predicted HRV reactivity and behavioral reactions, and negatively predicted maladaptive thoughts and negative affect. The finding that self-compassion promoted adaptive physiological and psychological responses relative to a recalled sport failure may have implications for performance enhancement, recovery, and health outcomes.

As well as positing and testing the imbuement of athletes with psychological skills in order to manage stress, some papers provide practical considerations for environmental factors that could aid adaptation to psychological stress. Hartley and Coffee test perceived availability of support and received support in regard to the main and stress-buffering effects of social dimensions of burnout. Data indicated that athletes who report greater levels of stress also reported higher burnout, and that higher levels of perceived availability of support was associated with lower levels of the burnout dimensions reduced sense of accomplishment and devaluation. Further, perceived availability of emotional support buffered the negative effects of high stress upon devaluation. The important role of support, and who provides it, is also illustrated in the work by Campo et al. on emotional intelligence (EI) training with the French u18 rugby union national. The aim of this study was to investigate the effectiveness of EI training programs provided by three different EI trainers, each of which has a support or leadership role in the team: the team's coach, the team's physiotherapist, and an expert in sport psychology. Linear mixed-effects models showed that the intervention helped the players to increase some emotional competences at the trait level highlighting the suitability of a group-based approach in the training-week structure and EI improvement in a short period of time. In terms of the broader environment Davis et al. examined the student-athlete experience of the dual career pathway. Surveys from 173 elite junior alpine skiers and interviews with six coaches also illustrated that optimizing support mechanisms across domains can promote positive adaptations to potential sources of stress.

As well as creating an environment in which athletes perceive high levels of availability of support, creating an adaptive motivational climate is also important. Ruiz et al. employed a two-wave approach to investigate the temporal interplay between motivation and the intensity and reported impact of athletes' emotions in training settings. They found that a higher task involving climate was related to decreased dysfunctional anxiety and dysfunctional anger, and in contrast, that a higher ego-involving climate was related to an increase in the intensity and reported impact of dysfunctional anger. The authors make clear the importance of a coach-created motivational climate and the importance of identifying high levels of controlled motivation to help athletes better adapt to psychological stress.

## Conclusions and the Way Forward

Clearly, the topic of adaptation to psychological stress in sport remains a vibrant, progressive, and multi-perspective area of study. This makes conducting research in this area challenging, and bringing together the threads of this research is complicated and requires nuance. What is evident, is that the papers included here are of high-quality and reflect great diversity across theoretical approaches, methodologies, analytic strategies, and scope. The topic of adaptation to psychological stress in sport has an exciting future, and we implore researchers to build on these works to develop and refine theory. We hope that practitioners make use of this work to inform their practice. A key step for this area is to ensure that research findings leap out of the laboratory into the hands of practitioners who can test theory at the coalface. To facilitate this process, we urge researchers to engage with practitioners in the designing and dissemination of their work, and to test theory at the elite level of sport.

## Author Contributions

MT created an early draft of Editorial which was then expanded upon by MJ and final edits and approval was given by the remainder of the authors. All authors contributed to the article and approved the submitted version.

## Conflict of Interest

The authors declare that the research was conducted in the absence of any commercial or financial relationships that could be construed as a potential conflict of interest.
